# Role of VEGF, Nitric Oxide, and Sympathetic Neurotransmitters in the Pathogenesis of Tendinopathy: A Review of the Current Evidences

**DOI:** 10.3389/fnagi.2016.00186

**Published:** 2016-08-09

**Authors:** Sebastiano Vasta, Alberto Di Martino, Biagio Zampogna, Guglielmo Torre, Rocco Papalia, Vincenzo Denaro

**Affiliations:** Department of Orthopaedic and Trauma Surgery, Campus Bio-Medico University of RomeRome, Italy

**Keywords:** tendons, tendinopathy, VEGF, nitric oxide (NO), nociceptive substance P (SP), neurotransmitter agents

## Abstract

Chronic tendinopathy is a painful common condition affecting athletes as well as the general population undergoing to tendon overuse. Although its huge prevalence, little is known about tendinopathy pathogenesis, and even cloudier is its treatment. Traditionally, tendinopathy has been defined as a lack of tendon ability to overcome stressing stimuli with appropriate adaptive changes. Histologic studies have demonstrated the absence of inflammatory infiltrates, as a consequence conventional antinflammatory drugs have shown little or no effectiveness in treating tendinopathies. New strategies should be therefore identified to address chronic tendon disorders. Angiofibroblastic changes have been highlighted as the main feature of tendinopathy, and vascular endothelial growth factor (VEGF) has been demonstrated as one of the key molecules involved in vascular hyperplasia. More recently, attention has been focused on new peptides such as Substance P, nitric oxide, and calcitonin gene-related peptide (CGRP). Those new findings support the idea of a nerve-mediated disregulation of tendon metabolism. Each of those molecules could be a target for new treatment options. This study aimed to systematically review the current available clinical and basic science in order to summarize the latest evidences on the pathophysiology and its effect on treatment of chronic tendinopathy, and to spread suggestions for future research on its treatment.

## Introduction

With the increasing number of amateur sport practitioners, a growing prevalence of tendinopathy has been recorded in the last few years in Europe and United States (Andia and Maffulli, 2015), affecting mainly Achilles tendon, rotator cuff, extensor tendons at the lateral epicondyle of the elbow and patellar tendon (Huang et al., 2004; Papalia et al., 2013). It has been described as an altered healing response of the tendon to stressful conditions (Papalia et al., 2013), including repetitive microtrauma, overloads, and acute and chronic injuries. The huge prevalence, the invalidating symptoms, the long time needed to return to activities, and the challenging management of chronic tendinopathies, rise concerns about the best treatment for these diseases. Apart from surgery, a prompt clinical benefit is usually achieved by bed rest, topic, or systemic drugs (including, NSAID's) (Crisp et al., 2008; Zhang et al., 2013), taping, cryotherapy, or modalities such as laser therapy and shockwaves (Steunebrink et al., 2013). However, an integrated treatment that considers the biologic pattern related to tendinopathy is far to be defined (Andia and Maffulli, 2015). At present, a growing effort in the scientific literature is aimed to understand the whole array of molecular and structural changes that are involved in the pathogenesis of tendinopathy. Despite the recent development and diffusion of biological therapies such as Platelet Rich Plasma (PRP) and mesenchymal stem cells, most surgeons ignore the basic science that underlies the molecular targets of these treatments.

At the end of the biological processes that lead to tendon tissue healing, we acknowledge the synthesis of collagen, which is achieved by an increase in the number and in the function of fibroblasts. This process has already been investigated in several studies (Connell et al., 2009; Clarke et al., 2011; Ahmad et al., 2012) reporting on the results of direct injection of dermal fibroblasts (Ahmad et al., 2012) or mesenchymal bone marrow cells (Ellera Gomes et al., 2012). Next to that, several other biologic items have been investigated, including the pathways of major molecules that have been found relevant in the pathogenesis of tendinopathy, either considering the role of vascular supply and innervation of the tendon. Specifically, concerning the vascular function, the molecules that have been investigated include the Vascular Endothelial Growth Factor (VEGF), the Hypoxia Inducible Factor (HIF), and the Nitric Oxide (NO); regarding the neurotransmitters, scientists mainly studied substance P (SP), Neurokinin-1 (N-1) and Calcitonin Gene-Related Peptide (CGRP). Several experimental studies aimed to stimulate the healing pathways acting directly on these molecular targets, showing promising results.

The aim of the present review of the literature is to explore the current literature regarding the role of vascular and neuronal molecular pathways in the pathogenesis and healing process of tendinopathy. Furthermore, the therapeutic implications of those pathways have been evaluated and shown in the below paragraphs.

## Materials and methods

**Figure 1:**
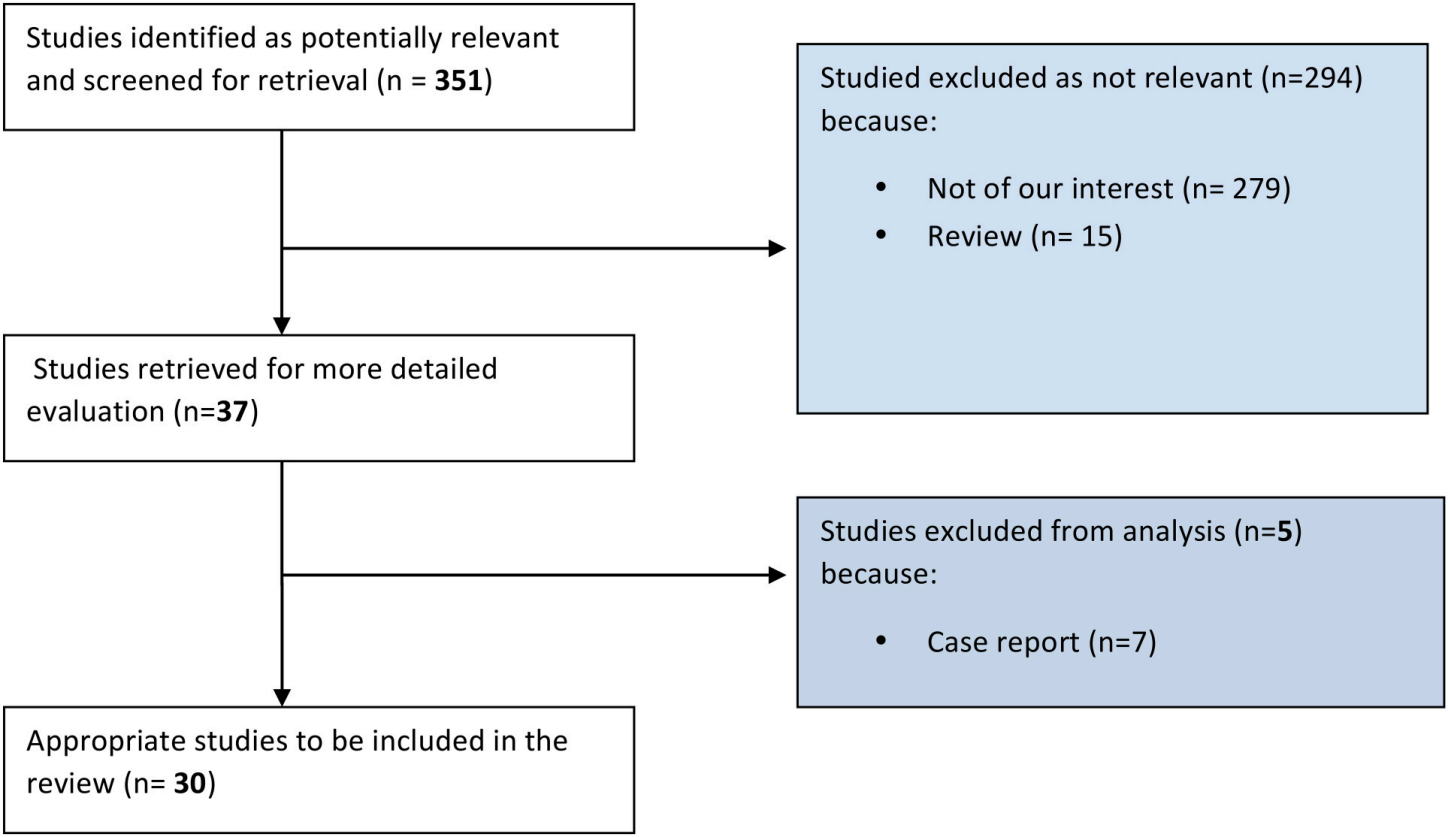
**Selection process of the studies**.

Articles research has been carried out using PubMed online database (http://www.ncbi.nlm.nih.gov/pubmed), between June and August 2015. The combinations of key-words used were the following: “(“tendinopathy”[MeSH Terms] OR “tendinopathy”[All Fields]) AND (“vascular endothelial growth factor a”[MeSH Terms] OR “vascular endothelial growth factor a”[All Fields] OR “vegf”[All Fields]),” “(“tendinopathy”[MeSH Terms] OR “tendinopathy”[All Fields]) AND (“substance p”[MeSH Terms] OR “substance p”[All Fields] OR “p substance”[All Fields]),” “(“tendinopathy”[MeSH Terms] OR “tendinopathy”[All Fields]) AND (“neurotransmitter agents”[Pharmacological Action] OR “neurotransmitter agents”[MeSH Terms] OR (“neurotransmitter”[All Fields] AND “agents”[All Fields]) OR “neurotransmitter agents”[All Fields] OR “neurotransmitter”[All Fields]).” The search was aimed to retrieve any level of evidence studies concerning molecular pathways involved in pathogenesis of tendinopathy, clinical associated features and therapeutic implications. Both clinical and experimental *in vivo* and *in vitro* studies were included. No study types were excluded except for literature reviews and case reports. No time interval for publication was set. Of each of the retrieved articles, the whole bibliography was carefully checked to enrich the research with possible studies relevant for the present work. Results of the studies were read, analyzed, and tabulated. The included studies have been divided into three categories: vascular function, nervous function, and therapeutic studies. The study selection process was carried out as shown in Figure 1.

## Results

### Vascular function (Table 1)

#### Behavior of endogenous angiogenetic factors in tendinopathy

Studies have demonstrated that neovascularization is one of the main features of tendinopathy (Zanetti et al., 2003; Rees et al., 2006) and that it is mainly a VEGF-driven process (Abraham et al., 2002; Yamazaki and Morita, 2006). In normal, asymptomatic adult tendons, the expression of VEGF is mostly suppressed, while in chronic overused tendons, VEGF expression is markedly increased in the early and late phases of the overuse process (Perry et al., 2005). Many *in vitro* studies have showed a close relation between cyclic strain and increased VEGF expression. In a recent study (Mousavizadeh et al., 2014), it has been found that cyclic strain applied to *in vitro* tendon cells yields to angiogenetic factors gene expression and synthesis, including angiopoietin like-4 (ANGPTL4), fibroblast growth factor-2 (FGF-2), cyclooxygenase-2 (COX-2), sphingosine kinase-1 (SPHK1), (transforming growth factor) TGF-α, VEGF-A, and VEGF-C. Comparable results were obtained by Petersen et al. (2004), that showed an increased synthesis of VEGF and Hypoxia Induced Factor-1α (HIF-1α) in fibroblasts cultures of rat tendons when an intermittent strain stress was applied. Nakama et al. (2006) found increased levels of VEGF and VEGFR-1 in tendons stimulated with continuous loading, compared to unstimulated tendons (*p* = 0.0001 and *p* = 0.046 respectively). An *in vivo* study on rabbits, by means of specific exercise protocols, Andersson et al. (2011b) showed an increase in tenocyte number and vascularization at 3 and 6 weeks. At the 6-week control, also the VEGF mRNA resulted overexpressed. Another *in vivo* study, by Sahin et al. (2012), investigated the expression of VEGF, HIF-1α, and MMP-3, and analyzed the tendons' biomechanical features in a tendinopathy model realized by freezing the rat's patellar tendon. At 7 days from the intervention, all the above-mentioned factors were increased and the angiogenesis was abundant. Furthermore, the biomechanical analysis showed a significant reduction in maximum stress and Young's module of the frozen tendons when compared with normal tendons. In human subjects, Scott et al. (2008), found that VEGF was overexpressed in patellar tendinopathy and not in normal tendons; moreover higher VEGF levels were detected in patients with symptoms of less duration (12 vs. 32.8 months). Chen et al. (2008) found postoperative increased levels of connective tissue growth factor (CTGF), TGF-β, VEGF, and Insulin-like growth factor-1 (IGF-1) after tendon repair in chicken models. Conversely, b-FGF expression was downregulated, and Platelet-Derived Growth Factor (PDGF) was slightly elevated. Consistently, Petersen et al. (2004) found an increase in VEGF levels after Achilles' tendon tenotomy in sheeps. In this model, the splice variants VEGF120 and VEGF164 were especially increased, at 3 and 24 weeks respectively. These findings demonstrate that VEGF is involved in the in mechanism of angiogenesis and Achilles' tendon repair.

**Table 1:** **Vascular function studies**.

**Study**	**Year**	**Investigated molecules**	**Type of study**	**Type of sperimentation**	**Role in pathogenesis**	**Role in healing**
Anitua et al.	2005	IGF-1, TGF-β-1, PDGF-AB, VEGF, HGF, EGF	*In vivo*	Comparison of the effect of platelet-poor and platelet-rich cloth releasates (PPCR, PRCR) and platelet-poor plasma (PPP) on tendon cell cultures		PRCR induces tenocytes proliferation more than PPCR
Mousavizadeh et al.	2014	ANGPTL4, FGF-2, COX-2, SPHK1, TGF-α, VEGF-A, and VEGF-C	*In vitro*	Strain protocol applied to tendon cell cultures. Measurement of gene expression and proteins synthesis	Tendon cell strain induces expression of angiogenetic growth factors	
Petersen et al.	2003	VEGF, HIF-1α	*In vitro*	Strain protocol applied to cell culture (3T3 fibroblasts and rat Achilles tendon cells) and measurement of factors expression	Cell strain increased VEGF and HIF-1α concentrations in culture supernatant	
Asundi et al.	2007	MMP-1, MMP-3, VEGF, CTGF, COX-2, IL-1β, COL-III, FBRN	*In vivo*	*In vivo* cumulative load of tendon vs. control and mRNA expression measurement	No difference of mRNA expression in loaded and unloaded tendons	
Sahin et al.	2012	VEGF, HIF-1α, MMP-3	*In vivo*	*In vivo* frozen model of tendinopathy was evaluated with immunohistochemistry		HIF-1α, VEGF, and MMP-3 increased after intervention, and then decreased after 14 and 28 days
Liang et al.	2012	HIF-1α, VEGF, Bnip3, Bcl-2, Bcl-xL	*In vitro*	Hypoxia protocol on tenocytes cultures. Measurement of HIF-1α, VEGF mRNA and pro- and anti-apoptotic peptides	Hypoxia induced overexpression of HIF-1α, VEGF, Bnip3, but not of Bcl-2 and Bcl-xL	
Hao Chen et al.	2008	CTGF, TGF-β, VEGF, IGF-1, bFGF, PDGF-B	*In vivo*	Surgical repair and post-operative harvesting of tendon on *in vivo* chicken model		CTGF TGF-β, VEGF. and IGF-1 were highly expressed during early healing period, bFGF was downregulated, and PDGF-B was minimally expressed
Andersson et al.	2010	VEGF mRNA	*In vivo*	Electrical *in vivo* stimulation and passive flexion of triceps surae on rabbit model. Tenocytes number, VEGF mRNA, and vascular density evaluation	Increased tenocyte number at 3 and 6 weeks of exercise. Increased vascularization in rabbits with 3-weeks exercise. VEGF mRNA increased at 6 weeks	
Petersen et al.	2003	VEGF, VEGFR-1, and VEGFR-2	*In vivo*	Tenotomy of Achilles tendon in *in vivo* sheep model. VEGF levels evaluation during healing		VEGF resulted increased in tenotomised tendons and not in healthy ones. Splice variants VEGF120 and VEGF164 were increased respectively at 3 and 24 weeks
Scott et al.	2008	VEGF	*In vivo*	Human patellar tendons with and without tendinosis were harvested. VEGF levels were assessed	VEGF was overexpressed in intimal cells of the vessels in affected tendon	
Nakama et al.	2006	VEGF, VEGFR-1, CTGF	*In vivo*	Cyclical loading applied to flexor digitorum profundus in rabbits. Expression of VEGF and receptor, CTGF were evaluated in loaded tendons and contralateral ones	VEGF, VEGFR-1, and CTGF were higher in loaded tendon, than in controls	
Sozomor et al.	2006	iNOS, eNOS	*In vivo*	Overuse protocol applied to supraspinatus tendon in animal model vs. controls, isoforms of the NOS measurement	eNOS and iNOS were four-fold increased when compared to controls	
Xia et al.	2006	iNOS, NO	*In vivo*	Achilles tendon repair in murine model iNOS^−/−^ vs. iNOS^−/−^ with aminoguanidine (iNOS inhibitor) vs. wildtype. Microscopic healing process evaluation and NO levels measurement		Healing process was comparable between iNOS^−/−^ and WT at 7 days from surgery. AG delayed healing in iNOS^−/−^, but NO levels were higher in WT group
Xia et al.	2006	NO, iNOS	*In vitro*	Tendon cells from rotator cuff were cultured and stimulated with NO and iNOS gene carrier. Enzimatic inhibition was performed		Low NO doses promoted fibroblasts proliferation, high NO doses inhibited proliferation
Lin et al.	2001	nNOS, iNOS, eNOS	*In vivo*	Achilles tendon surgery in rats and mRNA level of NOS isoforms measurement		All NOS isoforms were increased during healing

#### Behavior of endogenous nitric oxide in tendinopathy

Studies concerning nitric oxide (NO) and nitric oxide synthase (NOS) expression have been carried out, in order to evaluate endothelial activation during tendinopathy and its effects on tendon tissue. An overuse protocol applied to supraspinatus tendons was evaluated by Szomor et al. (2001), that found an increased expression of both inducible NOS (iNOS) and constitutive NOS (eNOS), compared to controls. The same authors found increased expression of iNOS and eNOS in human rotator cuff specimens harvested during rotator cuff repair surgeries (Szomor et al., 2001). However, NO seems to be important in new tissue synthesis during tendon healing. The NO-paracetamol association has been added to tendon tissue during *in vitro* Achilles tendon healing harvested from a murine model. Increased amount of collagen and improved collagen reorganization were found (Murrell et al., 2008). Murrell et al. (1997) showed a five-fold increase in NO synthase activity 7 days after surgical division of rat Achilles tendons, with levels approximating the baseline at day 14. Moreover, when the activity of NO synthase was inhibited, a significant reduction in cross-sectional area and in the load to failure of the tendons was observed. Lin et al. (2001) found similar outcomes after Achilles tendon surgery in rats, showing increased expression of all the isoforms of NOS. A study by Xia et al. (2006b) evaluated the role of iNOS in the healing process of tendinopathy from a murine model. Three groups were compared: group 1: wild-type (iNOS^+/+^); group 2: knock-out (iNOS^−/−^); and group 3: knockout (iNOS^−/−^) + systemic NO synthase inhibition through aminoguanidine (AG) administration. When systematically inhibiting the NO synthase in iNOS^−/−^ mice (group 3), the cross-sectional area of the healing Achilles tendon was significantly reduced. However, no significant differences were found between the wild-type (group 1) and the knock-out mice (group 2) concerning both the cross-sectional area and biomechanical features of the healing Achilles tendon. Moreover, the same authors (Xia et al., 2006a) demonstrated an increased collagen synthesis in cultures of human rotator cuff tenocytes when cells were exposed to exogenous NO (in the form of S-nitro-N-acetyl-penicillamine), and when these were transfected with the iNOS gene via an adenovirus vector.

### Nervous function and neuronal stimulation (Table 2)

#### Behavior of endogenous neurotransmitters in tendinopathy

It has been recently considered a role for neurotransmitters in the evolution of tendinopathy. The main molecule that has been investigated is Substance P (SP), which is known to play several roles in proliferation of fibroblasts, angiogenesis, and pain transmission (Andersson et al., 2011a). Other molecules that have been investigated include Acetylcholine (Ach) and its receptors, Neuropeptide Y (NPY) and its receptors, and Glutamate (Glu) and its receptors. A recent study by Fong et al. (2013) evaluated the effect of Ach on Achilles tendon cell cultures, showing an increased number of viable cells and proliferation, and a phosphorylation of the epidermal growth factor receptor (EGFR) and the extracellular signal-regulated kinases ERK1/2 that are responsible of the Ach effects. NPY and receptors Y1 and Y2 were investigated by Bjur et al. (2009), that demonstrated by immunohistochemistry an increased expression of Y1 and high levels of NPY in human Achilles tendinopathy cultures. The same Authors (Bjur et al., 2008) showed that tenocytes from tendinopathic Achilles displayed Tyrosine Hydroxylase (TH) positive immunoreactions and reactions for TH mRNA, in addition to α1-adrenoreceptors, showing therefore evidence of local catecholamine production, not only at the protein level but also at the mRNA level. The authors advanced the hypothesis that tenocytes produce catecholamines with a possible autocrine/paracrine effects and that they can respond to sympathetic transmitters. Adrenergic stimuli can have an influence on degenerative/apoptotic events but can also induce cell proliferation (Zhang and Faber, 2001). These findings are supported by the study of Danielson et al. (2007) that showed in addition to immunoreactions for TH and α1-adrenoreceptors, a positivity for α 2A-, and β1-adrenoreceptors and for neuropeptide Y. High levels of these receptors were also detected in the blood vessels' walls. The same Authors (Danielson et al., 2008) in a further study on biopsies from paratendinous area dorsal to the proximal patellar tendon of tendinopathic patients who underwent arthroscopy, confirmed the presence of the immunoreaction patterns and specifically demonstrated a marked immunoreaction for sympathetic markers in the small and large blood vessels surrounding the abnormal tendon tissue. Schubert et al. (2005) found in human Achilles tendinopathy samples nerve fibers positive to Nociceptive substance P (SP), and these were significantly increased together with an inflammatory infiltration of B and T lymphocytes. Backman et al. in two studies (Backman and Danielson, 2013; Backman et al., 2014) showed that SP reduces the Anti-Fas-induced apoptosis in healthy human tenocytes, and that this antiapoptotic effect of SP is mediated through NK-1 R and Akt-specific pathways. These findings support the role of SP in inducing the marked hypercellularity seen in tendinopathy. The same Authors found (Backman et al., 2011) in an animal model of Achilles tendon overuse, that already after 1 week of overloading, the SP levels were significantly elevated compared to a control group. Zhou et al. (2014) showed that the injection of exogenous SP in Achilles tendons of rats yielded to significantly increased proliferation of pluripotent tendon cells (PTCs). Moreover, the reverse transcription polymerase chain reaction (RT-PCR) showed that SP upregulated the expression of non-tenocyte genes but downregulated the expression of tenocyte-related genes. These findings indicate that SP is responsible for enhanced PTCs' proliferation, promotes non-tenocyte differentiation and plays an important role in the development of tendinopathy. In a study of Lui et al. (2010) after collagenase-induced tendinopathy of the patellar tendon, double stance duration was evaluated and then immunohistochemical evaluation was carried out. Increased SP and CGRP were found at 2 weeks, but not at 4 weeks and 8 weeks. Other peaks were found at 12 weeks and 16 weeks. The increased expression of SP and CGRP positively correlated with the duration of double stance. The presence of increased blood vessels and sympathetic nerve components in chronic patellar and Achilles tendinopathy was also confirmed by the studies of Andersson et al. (2007) and from Lian et al. (2006). Anderson and colleagues undertook a histological study of the ventral portion of tendinopathic Achilles tendons and demonstrated the presence of large and small arteries and nerve fascicles. The nerve fascicles contained sensory nerve fibers, positive for SP and CGRP staining, and sympathetic nerve fibers (Andersson et al., 2007). Lian and colleagues showed that chronic painful patellar tendons exhibited increased occurrence of sprouting nonvascular sensory, substance P–positive nerve fibers compared to a control group (Lian et al., 2006). In a study by Schizas et al. (2012), a relevant correlation was found between SP and glutamate receptors in human patellar tendons affected by exercise-related tendinopathy. The expressions of N-methyl-D-aspartate receptor type 1 (NMDAR1) and its phosphorylated form (P-NMDAR) were evaluated together with SP levels and mGlu receptors family levels. They found an increased expression of SP, NMDAR1, p-NMDAR1, and mGlutR5, when compared to controls. Furthermore, a significant co-localization of SP and NMDAR1 was found exclusively in tendinopathic tendons and not in healthy controls, suggesting a possible role of SP in stimulating NMDAR1. These findings are supported by previous studies such as those from Alfredson et al. (2001a,b), which found both free glutamate and glutamate NMDAR1 receptors in human Achilles' tendons from patients with chronic Achilles tendinopathy, and free glutamate and glutamate NMDAR1 receptors, but not PGE_2_, in patellar tendons with tendinopathy in respect to normal tendons.

**Table 2:** **Nervous function and neuronal stimulation studies**.

**Study**	**Year**	**Investigated molecules**	**Type of study**	**Type of sperimentation**	**Role in pathogenesis**	**Role in healing**
Fong et al.	2012	Ach, mAch-R, ChAT, VAchT	*in vitro*	Achilles tendon cells cultures treated with Ach. Immunohistochemical evaluation of cells immunopatterns and EGFR activation		Ach adimistration increase viable cells number, proliferation and activation of the EGFR and ERK1/2 pathways
Bjur et al.	2009	NPY and receptors Y1 and Y2	*in vitro*	Achilles tendon cell culture from human tendon with tendinosis. Immunohistochemical evaluation	Increased expression of Y1 receptor and NPY was found	
Schubert et al.	2005	SP	*In vivo*	Achilles tendon tissue with tendinosis vs. spontaneous rupture without tendinosis were examined microscopically	Increased number of SP positive fibers was found in tendinosis tendons, and not in spontaneous rupture	
Backman et al.	2011	SP, NK-1R	*In vivo*	Overuse protocol applied *in vivo* to rabbit Achilles tendons for 1, 3 or 6 weeks vs. controls	Increased SP expression at each time. SP and NK-1R were found in blood vessel walls. NK-1R was also found on tenocytes	
Lui et al.	2010	SP, CGRP	*In vivo*	Collagenase-induced tendinopathy in patellar tendons in rats. Double stance duration before sacrifice assessment and immunohistochemical evaluation		Increased SP and CGRP were found at 2 week, but not at 4 weeks and 8 weeks. A second peak was at 12 weeks and 16 weeks. SP and CGRP changes were consistent with double stance duration
Schizas et al.	2012	SP, NMDAR1, p-NMDAR1, mGluR1, 5, 6, and 7	*In vivo*	Immunohistochemical evaluation of human patellar tendon tissue with exercise-related tendinopathy	Increased expression of SP, NMDAR1, p-NMDAR1 and mGlutR5 was found in comparison with controls. Co-localization of SP and NMDAR1 was found	

Consistently with SP's potent effect on stimulating proliferation of fibroblasts and endothelial cells, Burssens et al. (2005) demonstrated as paratendinous injections of SP after operative repair of the Achilles tendon in rats, significantly enhanced tendon healing compared to controls. Similar results were achieved by other authors. Steyaert et al. (2010) reported that exogenous injections of SP yielded to enhanced angiogenesis and fibroblast proliferation, and boosted the endogenous substance P effects for fibroblast proliferation via an autocrine/paracrine stimulation, though SP didn't stimulate sensory nerve ingrowth. Bring et al. (2012), after depleting SP levels through Capsaicin in rats who underwent surgical transection of the Achilles tendon, showed that rats with higher residual SP levels developed improved tensile strength and stress at failure in the healing of Achilles' tendons. Carlsson et al. (2011) showed that substance P injections enhance tissue proliferation and regulate sensory nerve ingrowth on sutured rat's Achilles tendon, previously torn. The contrasting effects of Substance P could find a possible explanation thanks to the finding from a recent *in vitro* study by Zhou et al. (2014). These authors demonstrated that adding high-doses of SP (5.0 nmol) to patellar tendon tissue yielded to tendinopathic changes. Low doses of SP (0.5 nmol) boosted up the tenogenesis process compared with saline injection (control group) and the high-dose SP group. These findings suggest that SP has a possible dose-dependent effect: low doses could be advantageous for tendon healing, while high SP doses can be responsible for tendinopathic evolution.

### Effects of exogenous VEGF, NO, and SP on tendinopathy (Table 3)

Almost all the previously described factors have been investigated in experimental or clinical setting to assess the biological response to this therapy on tendinopathy.

**Table 3:** **Therapeutic studies**.

**Study**	**Year**	**Investigated molecules**	**Type of study**	**Type of sperimentation**	**Role in healing**
Lu et al.	2008	VEGF	*In vivo*	Partial patellectomy on *in vivo* rabbit model. Healing stimulation through LIPUS vs. controls. VEGF expression measurement	VEGF expression resulted more increased in LIPUS group than controls
Liang et al.	2012	HIF-1α, VEGF, Bnip3, Bcl-2, Bcl-xL	*in vitro*	Hypoxia protocol on tenocytes cultures. Measurement of HIF-1α, VEGF mRNA and pro- and anti-apoptotic peptides	Insulin or PRP showed protective effects on cell death
Kaux et al.	2014	VEGF111	*In vivo*	VEGF111 vs. saline injection in Achilles tendon after artificial lesion in murin model	Force for tendon rupture at 15 and 30 days was higher in VEGF111 group
Zhang et al.	2003	VEGF, TGF-β, PDGF, bFGF, IGF-1	*In vivo*	Transection and suture repair of Achilles tendon in murin model. VEGF injection at repair site vs. saline injection. TGF-β levels measurement	Tensile strength was higher in tendons treated with VEGF than controls in low terms. TGF-β was increased in VEGF group. PDGF, bFGF, IGF-1 were comparable
Paoloni et al.	2005	GTN	*In vivo*	Local application of GTN patch for elbow extensors tendinopathy vs. controls. Measurement of pain, tenderness, peak force and total work	Exogenous NO administration improve healing of the tendons
Paoloni et al.	2004	GTN	*In vivo*	Rehabilitation plus local application of GTN patch for Achilles tendinopathy vs. controls. Measurement of pain, tenderness, peak force, and total work	Exogenous NO administration improve healing of the tendons
Steunebrink et al.	2013	GTN	*In vivo*	Eccentric exercise plus GTN patch application on patellar tendon vs. controls (exercise only)	Exogenous NO administration improve healing of the tendons
Murrell et al.	2008	NO-paracetamol	*in vitro*	*in vitro* administration of NO-paracetamol association to tendon cell culture	Improved collagen production and reorganization
Burssens et al.	2005	SP	*In vivo*	SP (different doses) administration to rats after Achilles tendon repair vs. tiorphan and captopril vs. controls. Microscopic evaluation of fibroblasts proliferation, angiogenesis and collagen organization	In tendons treated with SP, fibroblasts proliferation was increased at 7 days, but no at further f.u. Similar evolution for angiogenesis and collagen orientation

The administration of the splice VEGF-111 in artificially injured Achilles tendon was investigated by Kaux et al. (2014), who showed a significantly higher strength-to-rupture of the tendons at 15 and 30 days. Similar results were obtained by Zhang et al. (2003), which found higher tendon strength in rats treated with VEGF injection after Achilles tendon rupture and repair, compared to the controls.

The use of NO has been investigated in several experimental and clinical settings on different types of tendinopathies, and it has been administrated clinically through a transdermal route. Paoloni and colleagues demonstrated the beneficial effects of glyceril trinitrate (GTN) patches on elbow extensors tendon (McCallum et al., 2011) and on Achilles' tendon (Paoloni et al., 2004), with improvement of pain and function at follow-up, especially in reducing tenderness and increasing muscle's peak force and total work. Comparable results were obtained by Steunebrink et al. (2013), evaluating GTN application plus eccentric exercise for patellar tendinopathy. Double-blinded randomized trials (RCTs) have been conducted to assess whether NO is useful in tendinopathy. Specifically, Paoloni et al. (2003) investigated 86 patients with chronic extensor tendinopathy at the elbow, undergoing to rehabilitation plus either topical NO application in the form of GTN patch or placebo. Those patients from the treatment group showed significantly reduced pain at 2 weeks, as well as reduced pain during activity, reduced tenderness at the lateral epicondyle at 6 and 12 weeks and increased wrist extensor mean peak force at 24 weeks. In a further study (McCallum et al., 2011) a subset of patients from the original trial was follow-upped at 5 years, and patients from the treatment group did not score better than those from the placebo group. In a double-blinded RCT on patients affected by Achilles tendinopathy, two groups were compared, either undergoing to rehabilitation plus either GTN patch or placebo. The treatment group demonstrated significantly reduced Achilles activity pain at 12 weeks, reduced night pain at 12 weeks, reduced tenderness at 12 weeks, decreased pain scores with the hop test at 24 weeks, and an increase in ankle plantar flexor mean total work at 24 weeks (Paoloni and Murrell, 2007). The same patients were further assessed at 3 years, and the results showed as 88% of patients with GTN treatment were asymptomatic at 3 years compared with 67% of patients treated with tendon rehabilitation alone (Paoloni and Murrell, 2007).

The SP administration in tendinopathy has been extensively studied by Burssens et al. (2005) who studied the effects of NO in surgically repaired Achilles tendon of rats. In the group receiving exogenous SP, increased fibroblasts proliferation, angiogenesis and collagen fibers organization were found at 7 days post-operatively showing a faster healing of Achilles tendons, even though there were no differences at 14 days with the control group. An interesting study from Mousavizadeh et al. (2015) showed that exposing human tendon cells to dexamethasone resulted in a time-dependent reduction of mRNA for SP. However, it should be taken into account as there is huge evidence that steroids negatively affect viability, migration, proliferation, and collagen synthesis of both human and animal tenocytes (Tsai et al., 2003; Wong et al., 2003, 2009). Zhang et al. (2013) in fact recently demonstrated that dexamethasone exposure induced non-tenocyte differentiation of human tendon stem cells, determining a nearly complete suppression of collagen type I expression, and an up-regulation of non-tenocyte related genes (PPARg and Sox-9). Whether, this mechanism may be mediated by an under-regulation of SP levels, has not yet been demonstrated.

## Discussion

The increasing knowledge concerning tendon dysfunction, clinically expressed as tendinopathy, leads to the individuation of a huge array of factors implied in the pathogenesis and repair mechanisms of this disease. Among single molecules and pathways involved in pathogenesis and healing process of tendinopathy, vascular, and neuronal factors play a major role (Papalia et al., 2013, 2015; Notarnicola et al., 2014). The first ones are mainly involved in angiogenesis and vascular activation, while the second ones concern signaling for cell proliferation, collagen organization and pain transmission (Alfredson et al., 2003). All these features are considered the core of the pathogenesis, although their proper timing through each step of the tendinopathy process still remains unclear. Some attempts have been performed to find out the exact time and way of activation of each specific pathway, quintessential to find a targeted therapy for the tendinopathy process. From a clinical point of view, tendinopathy represents an invalidating disease, with a severe impact not only on sport practice, but also on daily activities. The main challenge when facing with tendinopathy is to provide patients with a therapy that is effective and fast-acting and provides for durable outcome.

Some studies have investigated the role that VEGF has in both the pathogenesis and to the healing response of tendinopathy, also using VEGF and its splice variants as an efficient treatment (Zhang et al., 2003; Kaux et al., 2014), that resulted in fibroblast proliferation and angiogenesis stimulation (Kaux et al., 2014). It has been found that VEGF and its receptor are the earlier molecules expressed in tendinopathy (Nakama et al., 2006), and even though the vascular hyperplasia induced by VEGF may be considered a key factor in the pathogenesis of tendinopathy (Scott et al., 2008), it is also the main mechanism of healing to restore tissue integrity.

SP administration in surgically repaired rat tendons (Burssens et al., 2005), have also some advantages, especially in terms of quickness of action. In addition, SP should be considered the vault key that put together the vascular and neural function, since it is known that SP promotes vascular activation and vasodilatation, and also tissue hyperplasia (Zhou et al., 2014).

The local vascular activation is actually considered a still unclear chapter of the wide topic of tendinopathy. Studies have demonstrated an increased expression of NOS isoforms produced by endothelium in diseased tendons (Szomor et al., 2001). Conversely knock-out mice for iNOS did not show a severely impaired tendon healing response (Xia et al., 2006a). Moreover, it has been shown as NO has variable effects on tendon tissue depending on its concentration (Xia et al., 2006b). At present, transdermal administration of NO related drugs (namely GTN patches) have shown a positive short-term effect in tendon healing even in the clinical setting (Paoloni et al., 2003, 2004; Tsai et al., 2003; Mousavizadeh et al., 2015).

However, since VEGF and its pathways are the broadly known factors among a so complex pathogenesis, it should probably be the main factor to pay efforts on. An interesting research line is the regulation of VEGF action during the pathogenesis of tendinopathy (Lu et al., 2008; Andersson et al., 2011b; Kaux et al., 2014), which is assessing whether hypervascularization is truly beneficial for tendon healing, and if so, in what phases of the process. The possible answer to such contrasting data about each of the investigated molecule (VEGF, NO, SP,) could be the timing. Understanding the exact time of intervention of those molecules during the tendinophaty pathogenesis and the healing process could lead to a targeted and timed therapy to enhance healing.

## Conclusions

Vascularization and neuronal transmission play a key role in determining the pathogenesis of the tendinopathy. The mainly known factors implied in the process are VEGF, Substance P, and Nitric Oxide, although their exact role in the mechanism of tendinopathy is not well determined. More research should be carried out, especially studies involving human subjects, in order to assess the timing of action of those factors, to find out how therapies targeted to the phase of the disease process may fasten the healing process and the clinical recovery.

## Author contributions

AD, RP, and VD supervised the articles selection process and reviewed the final manuscript. SV, BZ and GT provided articles selection, manuscript writing, and table filling up.

### Conflict of interest statement

The authors declare that the research was conducted in the absence of any commercial or financial relationships that could be construed as a potential conflict of interest.
